# Leptospirosis in humans and selected animals in Sub-Saharan Africa, 2014–2022: a systematic review and meta-analysis

**DOI:** 10.1186/s12879-023-08574-5

**Published:** 2023-10-03

**Authors:** Jacob Mugoya Gizamba, Lawrence Mugisha

**Affiliations:** 1 Department of Wildlife and Aquatic Animal Resources, College of Veterinary Medicine, Animal Resources & Biosecurity, Makerere University, Kampala, Uganda; 2 Spatial Science Institute, University of Southern California, Los Angeles, USA; 3 Ecohealth Research Group, Conservation &Ecosystem Health Alliance, Kampala, Uganda

**Keywords:** Leptospirosis, Seroprevalence, Humans, Animals, Sub-Saharan Africa

## Abstract

**Background:**

Leptospirosis is an emerging neglected tropical zoonotic disease of public health importance causing substantial morbidities and mortalities among humans. The infection is maintained within the population through interactions between humans, animals, and the environment. Understanding the burden of disease in both humans and animals is necessary for effective prevention and control in Sub-Saharan Africa (SSA). Therefore, we aimed to determine the seroprevalence of leptospirosis in humans, selected domestic animals, and rodents in SSA.

**Methods:**

A comprehensive search was done in six databases: Scopus, PubMed, Google Scholar, CINAHL, Web of Science, and African Journals Online databases for articles published between 01 January 2014 and 30 August 2022. Thirty-seven articles distributed across 14 out of 46 countries in SSA were included. The random effects meta-analysis model was used to pool the extracted seroprevalence data.

**Results:**

The overall pooled seroprevalence of leptospirosis among humans was 12.7% (95% CI: 7.5,20.8), 15.1% (95% CI: 9.4,23.5), and 4.5% (95% CI: 0.4, 35.6) based on results obtained using ELISA, MAT, and PCR diagnostic methods respectively. The pooled seroprevalence estimates among cattle were 29.2%, 30.1%, and 9.7% based on ELISA, MAT, and PCR respectively. Further, the pooled seroprevalence in goats was 30.0% for studies that used MAT, and among rodents, the pooled seroprevalence estimates were 21.0% for MAT and 9.6% for PCR diagnostic criteria. The seroprevalence of leptospirosis varied extensively between studies, across SSA regions and study setting (rural or urban).

**Conclusion:**

Leptospirosis is widespread in SSA in both humans and animals based on the current results of the pooled seroprevalence in the limited studies available. The burden is high in animals and humans and underestimated due to limited studies and challenges with limited diagnostic capacity in most healthcare settings in SSA. Hence, we recommend that leptospirosis should be listed as a disease of concern and be included on the list of routine diagnostics among patients presenting with febrile illness in healthcare settings. Further, we recommend the enhancement of surveillance of leptospirosis in all countries in SSA and the development of strategies with a One Health perspective to effectively prevent and control leptospirosis.

**Supplementary Information:**

The online version contains supplementary material available at 10.1186/s12879-023-08574-5.

## Strengths and limitations of this study

- To the best of our knowledge, this is the first meta-analysis summarizing the prevalence of leptospirosis in both humans and animals in Sub-Saharan Africa.
- This review highlights the current extent of leptospirosis burden in humans and selected animals in different regions of Sub-Saharan Africa.
- Rigorous methodology and statistical techniques were employed to obtain precise pooled prevalence estimates.
- This review could be limited by the impossibility to disaggregate data based on the *Leptospira* serovar and serogroup because of limited diagnostic techniques available to isolate the exact *Leptospira* variants.

## Background

Leptospirosis is among the widely spread emerging zoonotic disease with epidemic potential and is considered by the World Health Organization (WHO) as a neglected disease [1]. The disease is caused by infection with the pathogenic strains of a bacterium called *Leptospira*, with more than 300 pathogenic serovars known worldwide [2, 3]. Leptospirosis has a ubiquitous distribution in nature, though it's most prevalent in tropical and humid climates due to favorable environmental conditions for the pathogen to thrive. Previous reports from mathematical modeling estimated the global annual incidence of leptospirosis to be 14.8 cases per 100,000 population with approximately over one million cases and 60,000 deaths annually [4]. The prevalence of the human disease is hyperendemic mostly in the Caribbean and Latin America, India, Southeast Asia, Oceania, and sub-Saharan Africa [1, 5]. However, some temperate regions such as Greece, Germany, France, and the Netherlands experience some endemicity to a lesser extent [5, 6].

Among humans, exposure to pathogenic *Leptospira* could either be through direct or indirect contact [7]. Direct transmission occurs when susceptible human’s mucous membrane gets into contact with pathogen-contaminated urine, tissues, and any organs of infected animals [8]. Indirect transmission occurs when humans get into contact with contaminated environment such as soil and water. The transmission tends to vary based on setting, whereby in rural areas, the transmission of pathogenic *Leptospira* is mainly driven by rainfall, livestock or wild animal close contact, and farming [9]. Whereas in urban settings, transmission among humans is largely perpetuated by rodent infestation, poor hygiene, and overcrowding, mainly occurring typically in urban slums of low and middle-income countries (LMICs) [10]. Natural disasters such as heavy rainfalls and flooding have also been associated with leptospirosis outbreaks among humans globally, though not always [9, 11, 12].

Among animals, *Leptospira* transmission occurs either directly through a susceptible animal getting into contact with infected urine or body fluids of another infected animal or indirectly through contact with contaminated water, vegetation, or soil [13, 14]. The environment is an important medium in the transmission cycle of *Leptospira* pathogens both in humans and animals [15]. As in humans, rodents are associated with massive outbreaks of leptospirosis in livestock populations in urban areas [16]. While in rural settings, outbreaks are commonly linked to animal breeding practices and extreme seasonal factors such as heavy rains, and flooding. Given the increase in leptospirosis outbreaks worldwide, and the interconnectedness between humans, animals and the environment, more research is needed to decipher the epidemiology, and ecology of the infection [5, 11, 17].

A recent systematic review of articles published till January 2014 on the prevalence of leptospirosis among humans in SSA indicated that data about occurrence of the infection is limited for many countries with some counties mostly those in central Africa having outdated data [6]. Another systematic review covering studies published between January 1930 and October 2014 reported the prevalence of human leptospirosis ranging between 2.3% and 19.8% in hospitalized patients in Africa [17]. While the prevalence in animals was reported to vary widely based on the target animal species and the diagnostic method used. In this review, the overall *Leptospira* infection prevalence in Africa among rodents by PCR ranged from 11.0% to 65.8% while among cattle tested by culture, the prevalence ranges from 1.1% to 10.4% of the sampled animals. No further review has been conducted since 2014 about the prevalence of *Leptospira* infection among humans and selected animal species in SSA.

To address these knowledge gaps, in the current understanding of human and animal *Leptospira* infection in SSA, a systematic review and meta-analysis of peer-reviewed articles published between 2014 and 2022 was performed following the PRISMA guidelines and checklist. The review aimed at addressing the following objectives (a) to determine the overall seroprevalence of leptospirosis in humans and selected animals in SSA between 2014 and 2022 and (b) to summarize the seroprevalence of leptospirosis in humans and animals based on SSA regions, diagnostic method, and study setting (rural vs urban).

## Methods

### Study design

The methodology of this systematic review and meta-analysis was guided by the Centers for Reviews and Dissemination guidelines [18], and the Preferred Reporting Items for Systematic Reviews and Meta-Analyses (PRISMA) guidelines [19]. The review was registered in the PROSPERO International Prospective Register of Systematic Reviews on 14^th^ July 2022 (CRD42022345844).

### Eligibility criteria

We included peer-reviewed studies, both observational and experimental studies that reported on the seroprevalence or had sufficient data to estimate the seroprevalence of leptospirosis among humans, cattle, goats, and rodents across the SSA region. The inclusion and exclusion criteria are outlined below.

#### Inclusion criteria

- Participants/ study subjects: humans, cattle, goats, and rodents in SSA
- Study designs: both observational (cross-sectional, case–control, cohort, or retrospective studies) and experimental studies (Randomized Control Trials, RCTs)
- Outcome type: leptospirosis, *Leptospira*l antibodies
- Measure of outcome: prevalence/ seroprevalence, or sufficient data to estimate the prevalence.
- Publication period: January 2014 to August 2022.

#### Exclusion criteria

- Study designs: reviews, mathematical modeling studies, case series, and qualitative studies
- Studies with no primary data or clear description of the methodology
- Studies conducted outside SSA.
- Published before January 2014

#### Leptospirosis case definitions

The following definitions were used for the diagnostic criteria for leptospirosis.

Among humans, clinical signs, and symptoms consistent with leptospirosis and any one of the following:

#### Confirmed cases

- Four-fold increase in microagglutination test (MAT) titer in acute and convalescent serum samples.
- MAT titer ≥ 1:400 in single or paired serum samples
- *Leptospira* DNA detected by polymerase chain reaction (PCR).

#### Probable cases

- Presence of IgM antibodies by enzyme-linked immunoassay (ELISA) or dipstick
- Presence of IgM/IgA antibodies in the immunofluorescence assay
- MAT titer ≥ 1:100 in a single acute-phase serum sample

Among the animals, the test results from either the ELISA, MAT, or PCR diagnostic techniques were considered to determine whether the animal was positive for *Leptospira* antibodies or leptospirosis infection.

## Data collection procedures

### Search strategy

A comprehensive search was conducted in the following databases for articles published on leptospirosis and *Leptospira*l antibodies among humans and animals in SSA between 01 January 2014 and 30 August 2022: Scopus, PubMed, Google Scholar, CINAHL, Web of Science, and African Journals Online. A search strategy that employs medical subject headings (MESH) and keywords were developed and used while searching for literature. We categorized the search terms according to geographic location (SSA); participants (humans and animals), and outcome of interest (seroprevalence of leptospirosis). The final search strategy for PubMed was reported (Supp Table 1). The search terms used in PubMed were adopted and used in other databases. Furthermore, a search of the reference lists of the eligible papers was conducted to obtain other relevant articles.

### Selection of sources of evidence

Identified articles were uploaded into EndNote to remove duplicates. The articles were then screened based on title and abstract for eligibility by one author (JMG). The full texts of studies selected after screening were retrieved and screened to verify their conformance with the inclusion criteria. This process of screening was conducted by one author (JMG) and reviewed by the second author (LM).

### Data extraction process

For all included studies, data were extracted using a customized data abstraction tool designed for this study. The following information was extracted:

- Authors: Name of the first author and publication year
- Characteristics of the study: study title and objective, country, setting (urban/ rural), study design, data collection period (year, season), sample size, and diagnostic methods used.
- Participants: humans: age (range, mean, median), sex
- Outcome characteristics: number of leptospirosis cases, seroprevalence of leptospirosis
- Animals: species (cattle, goat, rodent), leptospirosis cases, sample size

Where data such as sample size and number of human participants or animals that were seropositive were provided, the seroprevalence estimates were calculated using this data.

## Statistical methods

### Data synthesis

Data were analyzed using the meta package in R software (version 3.6.1). Forest plots were drawn to visualize the pooled seroprevalence and the 95% confidence intervals (95% CI) of leptospirosis in humans and animals in SSA. A random effects meta-analysis model was used to pool the seroprevalence data [56]. Heterogeneity was assessed using the χ2 test on Cochrane’s Q statistic [57] and the I^2^ (values of 25%, 50%, and 75% representing low, medium, and high heterogeneity, respectively) [58]. Subgroup analysis based on the SSA region, diagnostic methods, and study setting (rural/urban), was done for studies that involved human participants. As a result of fewer animal studies, the pooled prevalence estimates of leptospirosis were categorized only based on the diagnostic criteria used and the study setting (rural vs urban setting).

### Risk of bias and quality assessment

The included articles were evaluated for methodological quality using a 10-item scale developed by Hoy et al. [59] for internal and external validity, generalizability, and response rate. The risk of bias assessment was conducted by JMG and reviewed by LM. The results of risk of bias (ROB) were presented for each study (Table 1).

**Table 1:** Summary characteristics of included studies

SSA region	country	Study setting	study population (sample size)	diagnostic criteria	ROB	reference
CA	DRC	urban and rural	humans (1300)	ELISA	Low	[20]
	DRC	rural	humans (38)	PCR	Moderate	[7]
	DRC	rural	humans (54)	MAT	Low	[21]
	CAR	urban and rural	humans (198)	ELISA	Moderate	[22]
	CAR	urban and rural	humans (497)	ELISA, MAT	Moderate	[23]
EA	Rwanda	Urban and rural	humans (377)	ELISA, MAT	Low	[24]
	Tanzania	urban	humans (250)	MAT	Low	[25]
	Kenya	Urban and rural	humans (737)	ELISA	Low	[26]
	Tanzania	Urban and rural	humans (370)	ELISA, MAT	Low	[27]
	Tanzania	rural	humans (267), Cows (1103), goats (248), rodents (207)	MAT	Low	[28]
	Tanzania	Urban and rural	humans (1225)	MAT	Low	[29]
	Tanzania	rural	humans (455), rodents (24)	MAT	Low	[30]
	Tanzania	Urban and rural	humans (1293)	MAT	Low	[31]
	Uganda	rural	humans (359)	MAT	Low	[32]
	Tanzania	rural	humans (191)	PCR	Low	[33]
	Tanzania	rural	humans (842)	PCR	Low	[34]
	Tanzania	urban	humans (205)	MAT	Low	[35]
	Tanzania	rural	humans (267)	MAT	Low	[36]
	Tanzania	rural	humans (50), goats (45), rodents (45)	MAT	Low	[37]
	Tanzania	rural	humans (128)	PCR	Low	[38]
	Uganda	urban	humans (254)	MAT	Low	[39]
	Mozambique	rural	humans (373)	ELISA, MAT	Low	[40]
	Kenya	rural	cows (1170)	ELISA	Low	[41]
	Kenya	rural	cows (415)	ELISA	Low	[42]
	Tanzania	rural	cows (452), goats (162), sheep (89), rodents (384)	PCR	Low	[43]
	Uganda	urban and rural	cows (500)	PCR	Low	[44]
	Uganda	rural	cows (92)	ELISA	Low	[45]
	Tanzania	rural	rodents (89)	MAT	Low	[46]
	Mozambique	urban	rodents (57)	PCR	Low	[47]
SA	South Africa	rural	humans (138)	ELISA	Low	[48]
	South Africa	rural	cows (199)	MAT	Low	[49]
WA	Ghana	rural	humans (657)	ELISA	Low	[50]
	Sierra Leone	urban and rural	humans (100)	PCR	Low	[51]
	Senegal	rural	humans (545), cows (56), goats (52), sheep (43)	MAT	Low	[52]
	Burkina Faso	urban and rural	humans (781)	ELISA, MAT, PCR	Low	[53]
	Ivory Coast	urban and rural	humans (384)	ELISA, MAT	Low	[54]
	Nigeria	urban and rural	cows (190)	MAT, Culture	Low	[55]

*SSA* Sub-Saharan Africa, *ROB* Risk of bias, *CA* Central Africa, *EA* East Africa, *SA* Southern Africa, *WA* West Africa, *MAT* Mat agglutination test, *ELISA* Enzyme-linked immunoassay, *PCR* Polymerize chain reaction, *DRC* Democratic republic of Congo

### Publication bias assessment

The funnel plots and Egger’s weighted regression methods [60] were used to assess publication bias and a *p*-value < 0.10 was considered indicative of statistically significant publication bias. Funnel plots are presented in the supplementary files.

## Results

### Study selection

**Fig. 1:**
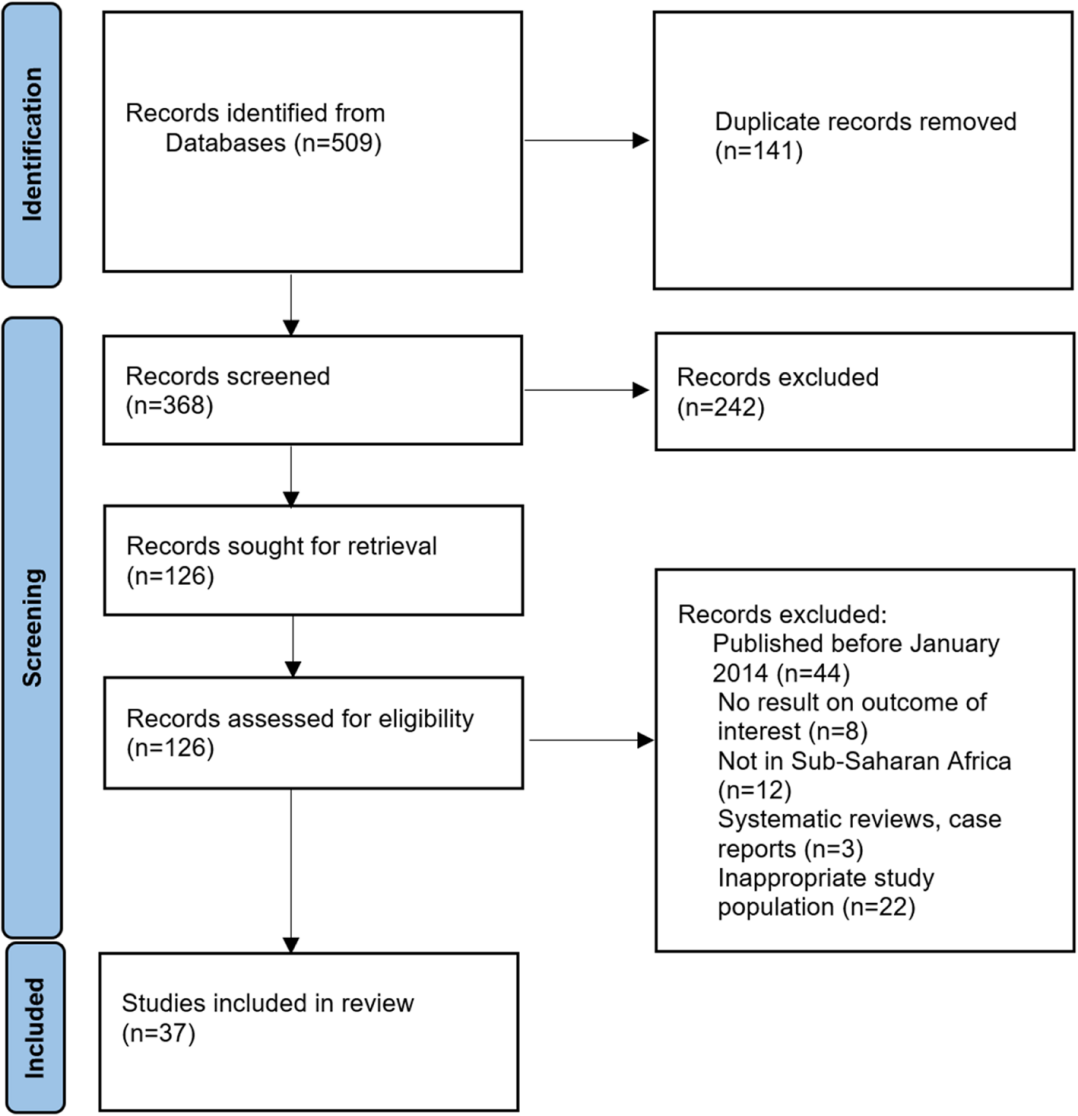
Flowchart of study selection

We found 509 articles in the literature search. When duplicates (*n* = 141 articles) were removed, 368 articles remained for both abstract and full-text screening. In the title and abstract screening, 242 articles were excluded because they did not meet the inclusion criteria (Fig. 1). The full-texts of the remaining 126 articles were obtained and after full-text screening, 89 articles were excluded because they didn’t meet the inclusion criteria. Finally, 37 articles were included in the systematic review. To report the seroprevalence of leptospirosis, PCR and culture results reported by studies that used these techniques to test for leptospirosis were excluded while conducting the meta-analysis.

### Summary of included studies

**Fig. 2:**
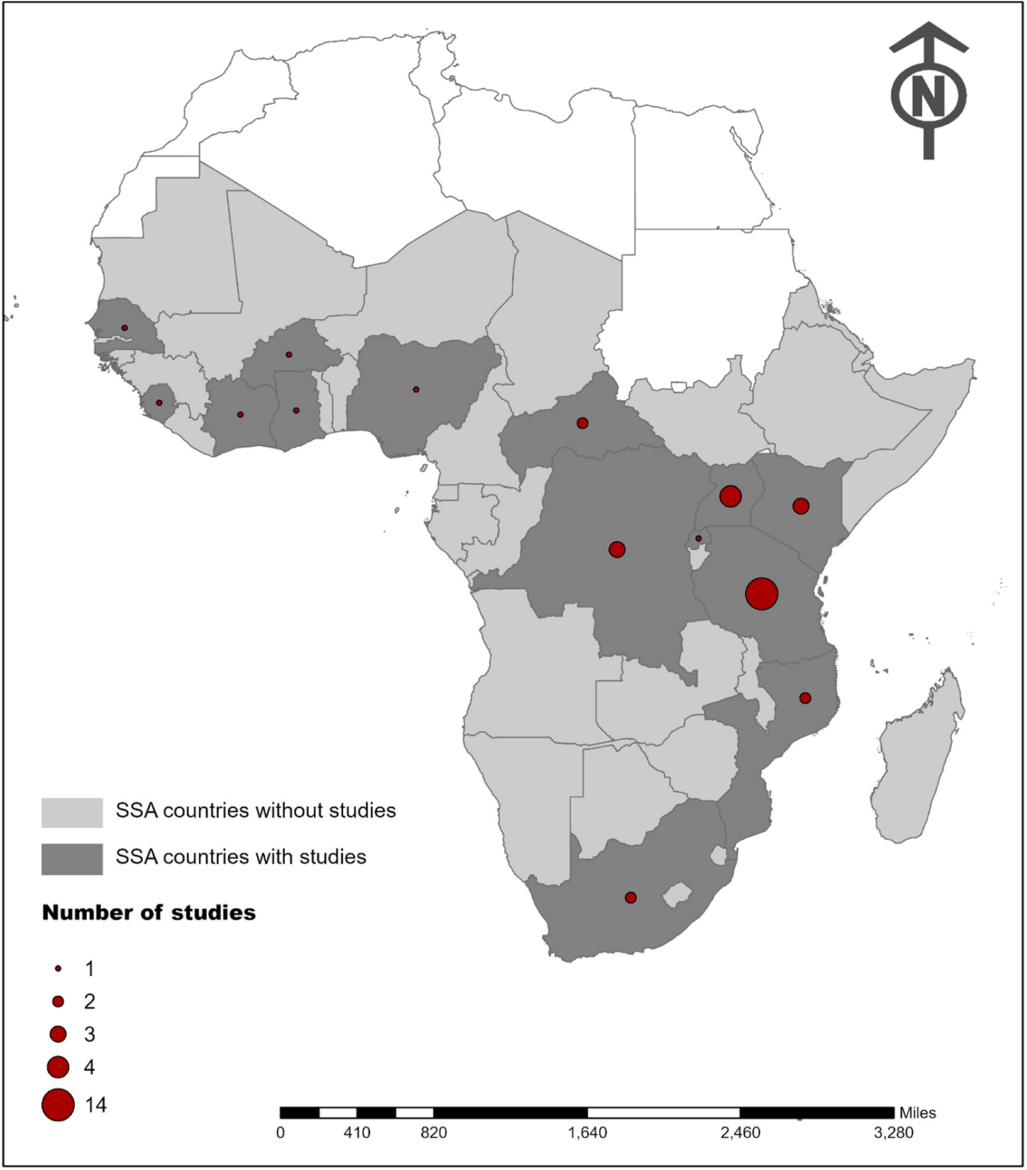
Distribution of studies on Leptospirosis from countries in the SSA between 2014 and 2022

Thirty-seven articles from 14 countries out of 46 countries in SSA were included in this review. Most of the studies (*n* = 24, 64.9%) were conducted in the East Africa (EA) region particularly in Tanzania (*n* = 14) (Fig. 2). The studies conducted in Central Africa (CA), West Africa (WA), and Southern Africa (SA) regions were 5 (13.5%), 6 (16.2%), and 2 (5.4%) respectively (Table 1). All the included studies used a cross-sectional study design, with 20 (54.1%) studies conducted particularly in a rural setting, 4 (10.8%) studies in an urban setting, and 13 (35.1%) studies conducted in both urban and rural settings. The most used diagnostic method was the MAT (*n* = 21, 56.8%) used either alone or in combination with other methods such as ELISA or culture or PCR. The studies that used the ELISA method alone were 8 and the studies that used the PCR method alone were also 8 (Table 1). All of the included studies had a low ROB (*n* = 34, 91.9%) except 3 (8.1%) that had a moderate ROB.

### Prevalence of *Leptospira* in humans

**Fig. 3:**
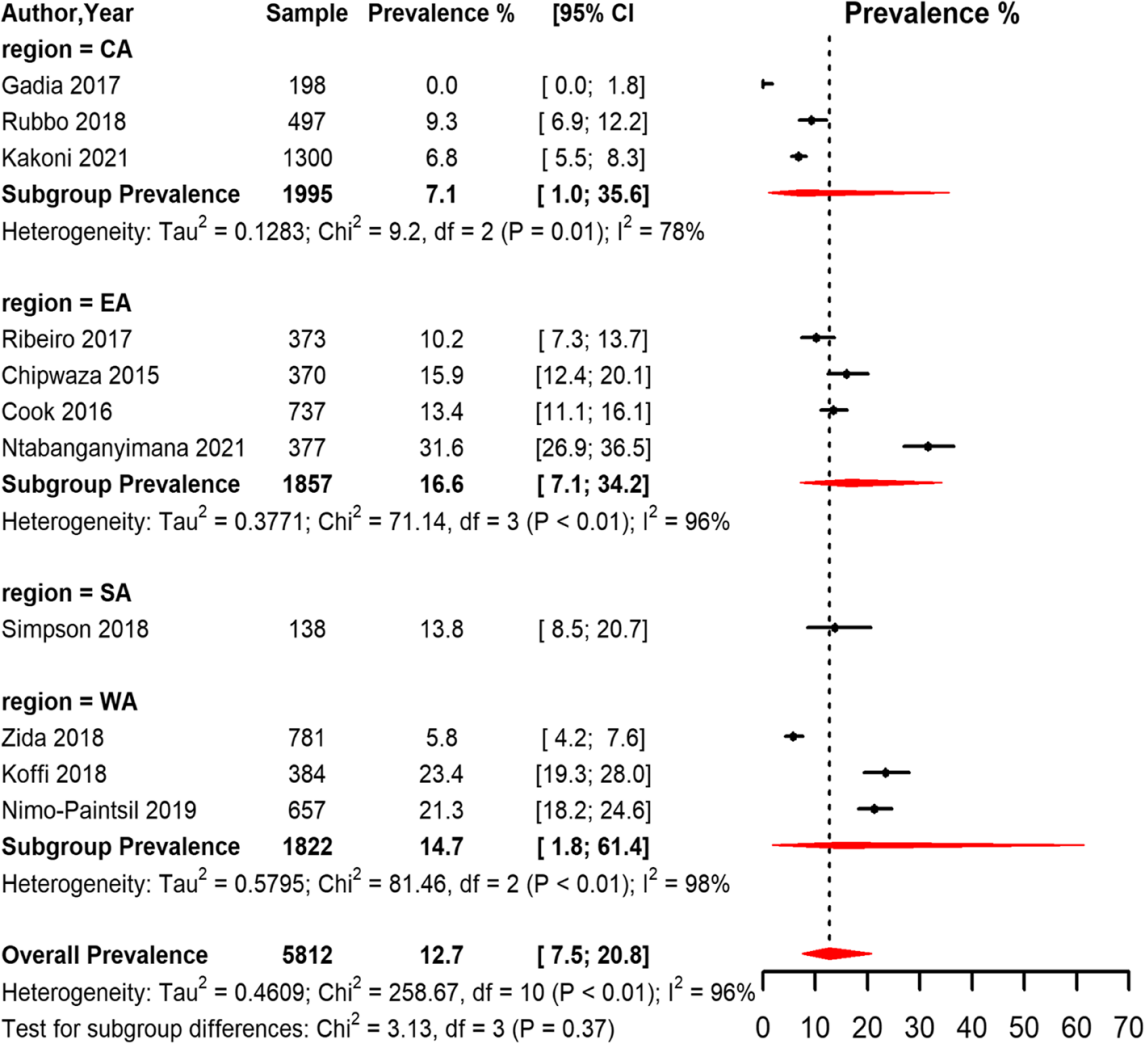
Forest plot of the seroprevalence estimates of Leptospirosis by ELISA method in humans across SSA regions. (CA: Central Africa; EA: East Africa; SA: Southern Africa; WA: West Africa CI: confidence interval. The vertical dotted line represents the overall prevalence, and the red diamond represent the pooled prevalence for each region)

Based on the ELISA method, the overall seroprevalence of leptospirosis across the regions in SSA was 12.7% (95% CI 7.5,20.8) with substantial heterogeneity between studies (I^2^ = 96.0%, *p* < 0.01) (Fig. 3). Based on the funnel plot (supp Fig. 1) and Egger's test, there was symmetry and no evidence of potential publication bias. The EA region had the highest pooled seroprevalence compared to other regions in studies that used the ELISA method.

**Fig. 4:**
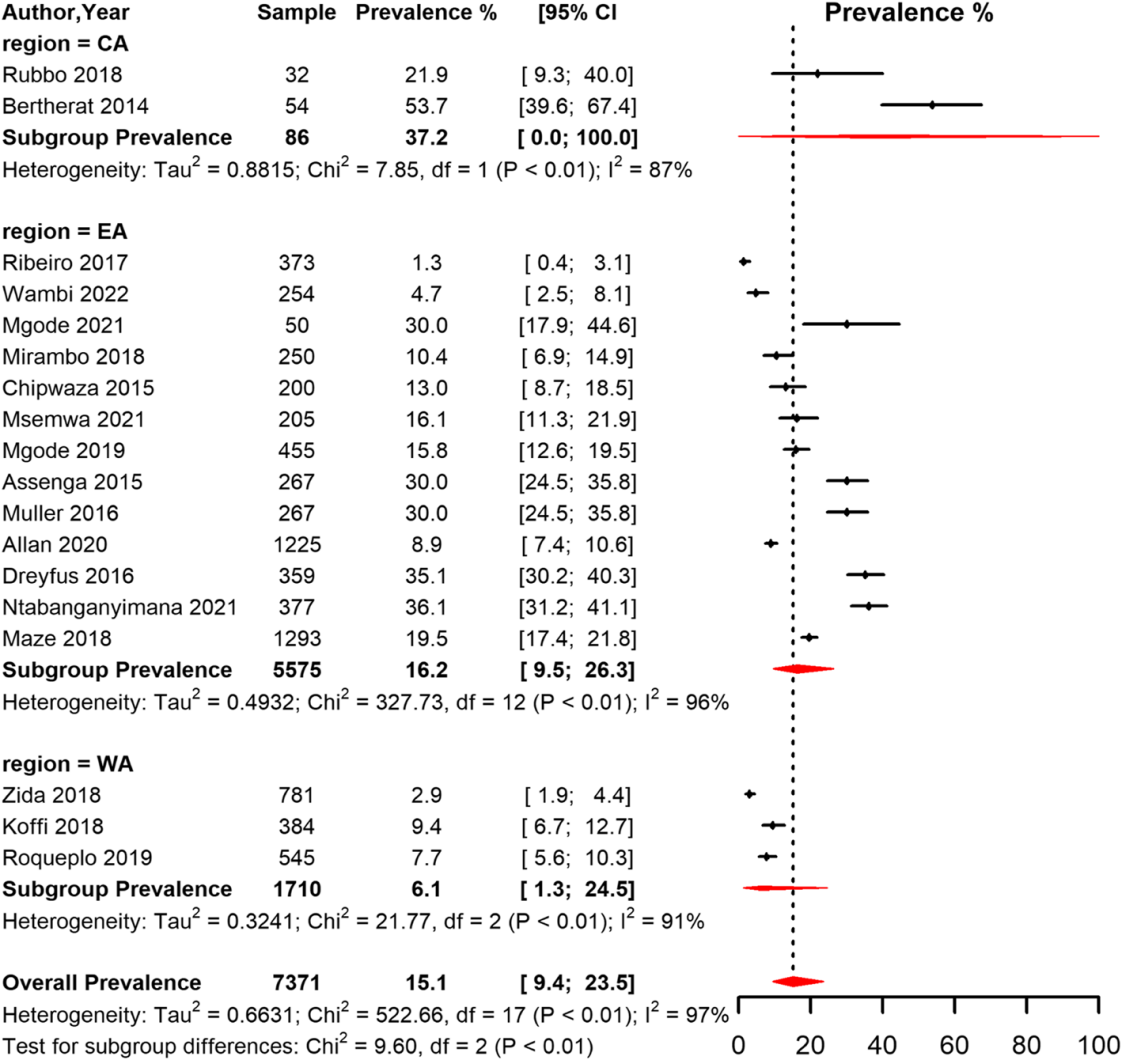
Forest plot of the seroprevalence estimates of Leptospirosis by MAT method in humans across SSA regions. (CA: Central Africa; EA: East Africa; WA: West Africa CI: confidence interval. The vertical dotted line represents the overall prevalence, and the red diamond represent the pooled prevalence for each region)

Based on the MAT method, the overall seroprevalence of leptospirosis was 15.1% (95% CI 9.4,23.5) with substantial heterogeneity between studies (I^2^ = 97.0%, *p* < 0.01) (Fig. 4). Based on the funnel plot (supp Fig. 2) and Egger's test, there was symmetry and no evidence of potential publication bias. CA region had the highest pooled seroprevalence compared to other regions among studies that used MAT diagnostic method. Based on the PCR method, the overall seroprevalence of leptospirosis across the regions in SSA was 4.5% (95% CI 0.4, 35.6) (supp table 2). When studies were sub-grouped based on whether they were conducted in a rural or urban setting, the pooled seroprevalence of studies conducted in rural areas was higher than the pooled estimate for studies conducted in urban or a mixture of both urban and rural areas (supp Figs. 3 and 4).

### Prevalence of *Leptospira* in animals (cattle, goats, and rodents)

**Fig. 5:**
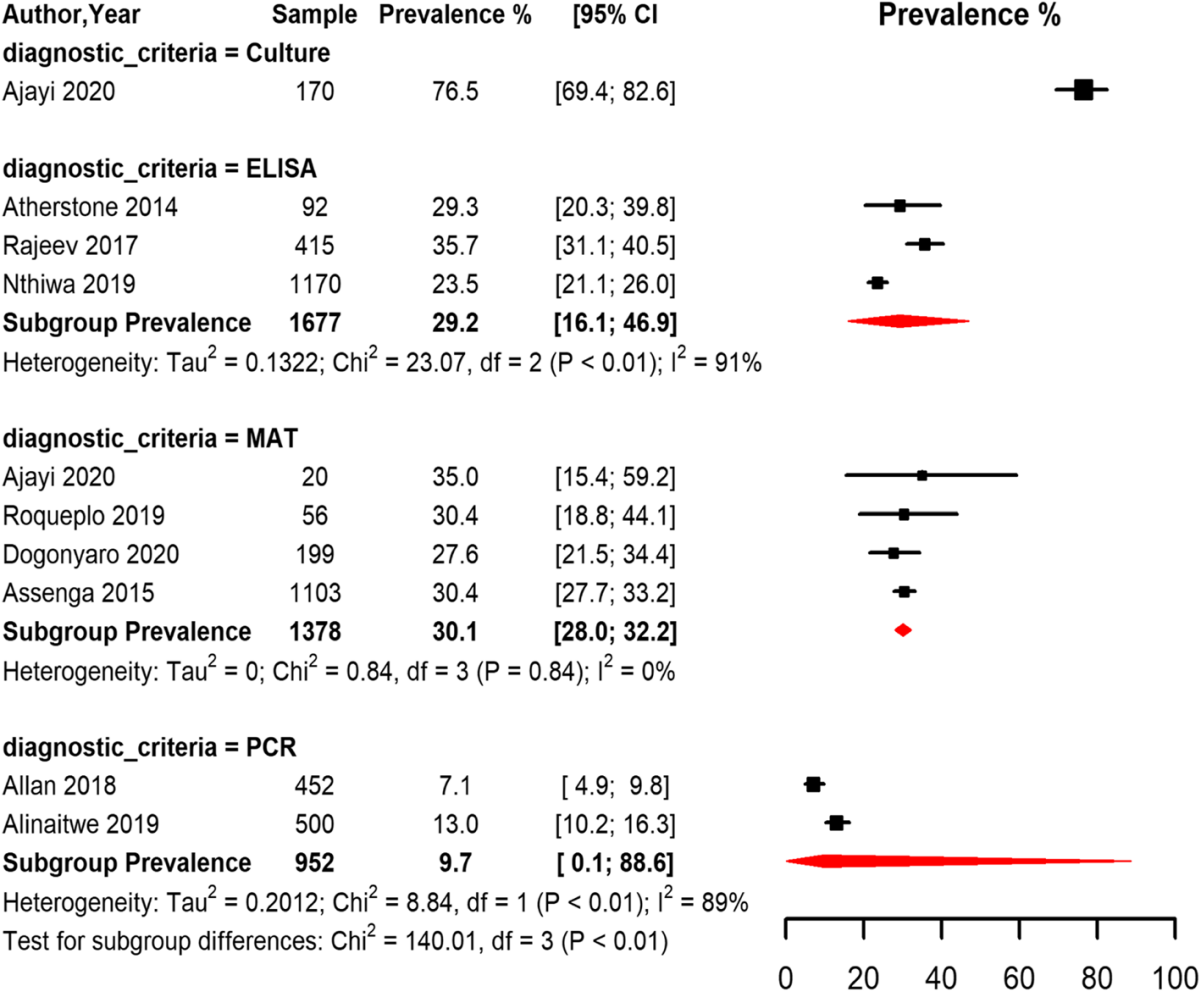
Forest plot of the seroprevalence estimates of Leptospirosis in cattle across SSA grouped by the diagnostic method. (MAT: Mat agglutination test; ELISA: Enzyme-linked immunoassay; PCR: polymerize chain reaction; CI: confidence interval. The red diamond represents the pooled prevalence for each diagnostic test)

Most of the studies that estimated the prevalence of *Leptospira* in cattle used MAT and ELISA methods, with only 2 studies and 1 study using PCR and culture methods respectively. Overall, the seroprevalence of leptospirosis in cattle was 29.2% (95% CI 16.1,46.9), 30.1% (95% CI 28.0,32.2), and 9.7% (95% CI 0.1,88.6) based on the ELISA, MAT, and PCR methods respectively (Fig. 5). The pooled seroprevalence estimated by the MAT method was higher compared to ELISA and PCR methods and the pooled seroprevalence differed significantly between the diagnostic methods (*p* < 0.01) (Fig. 5).

**Fig. 6:**
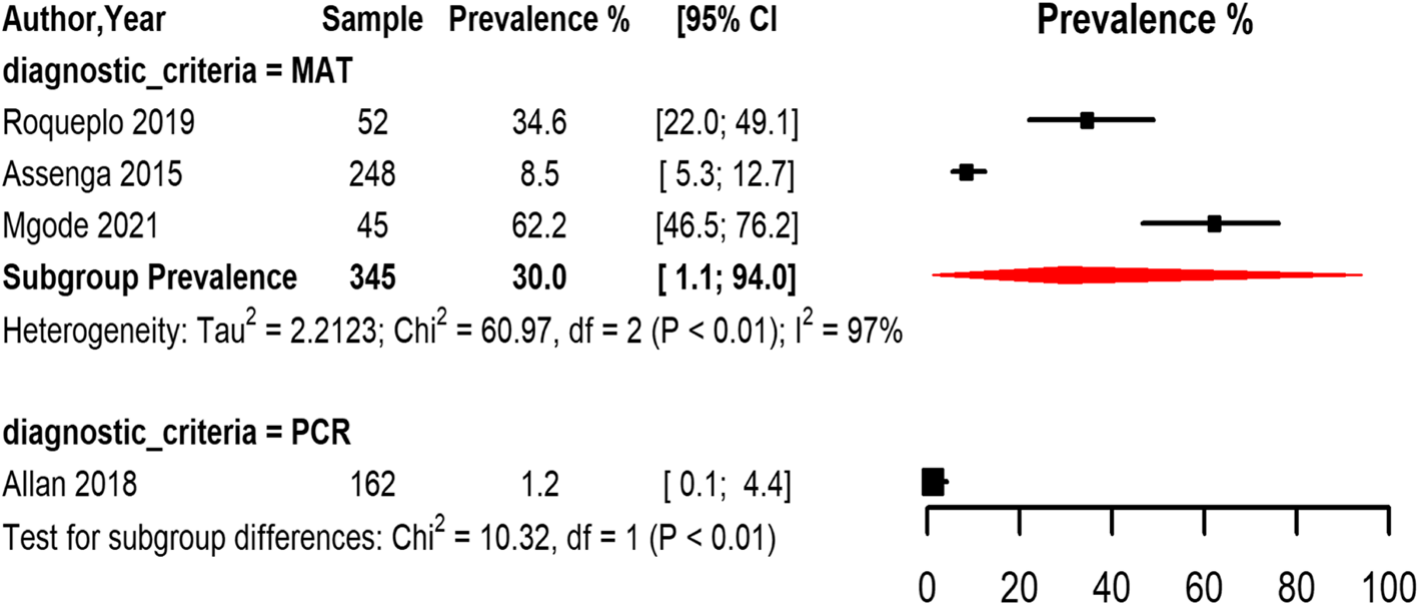
Forest plot of the seroprevalence estimates of Leptospirosis in goats across SSA regions grouped by the diagnostic method. (MAT: Mat agglutination test; PCR: polymerize chain reaction; CI: confidence interval. The red diamonds represent the pooled prevalence for each diagnostic test)

Most of the studies that estimated the seroprevalence in goats used the MAT diagnostic method, with only one study using the PCR method and no study using ELISA. The pooled seroprevalence estimates of *Leptospira* infection in goats among studies that used the MAT method was 30.0% (95% CI 1.1,94.0) with substantial heterogeneity among the studies (I^2^ = 97.0%, *p* < 0.01) (Fig. 6).

**Fig. 7:**
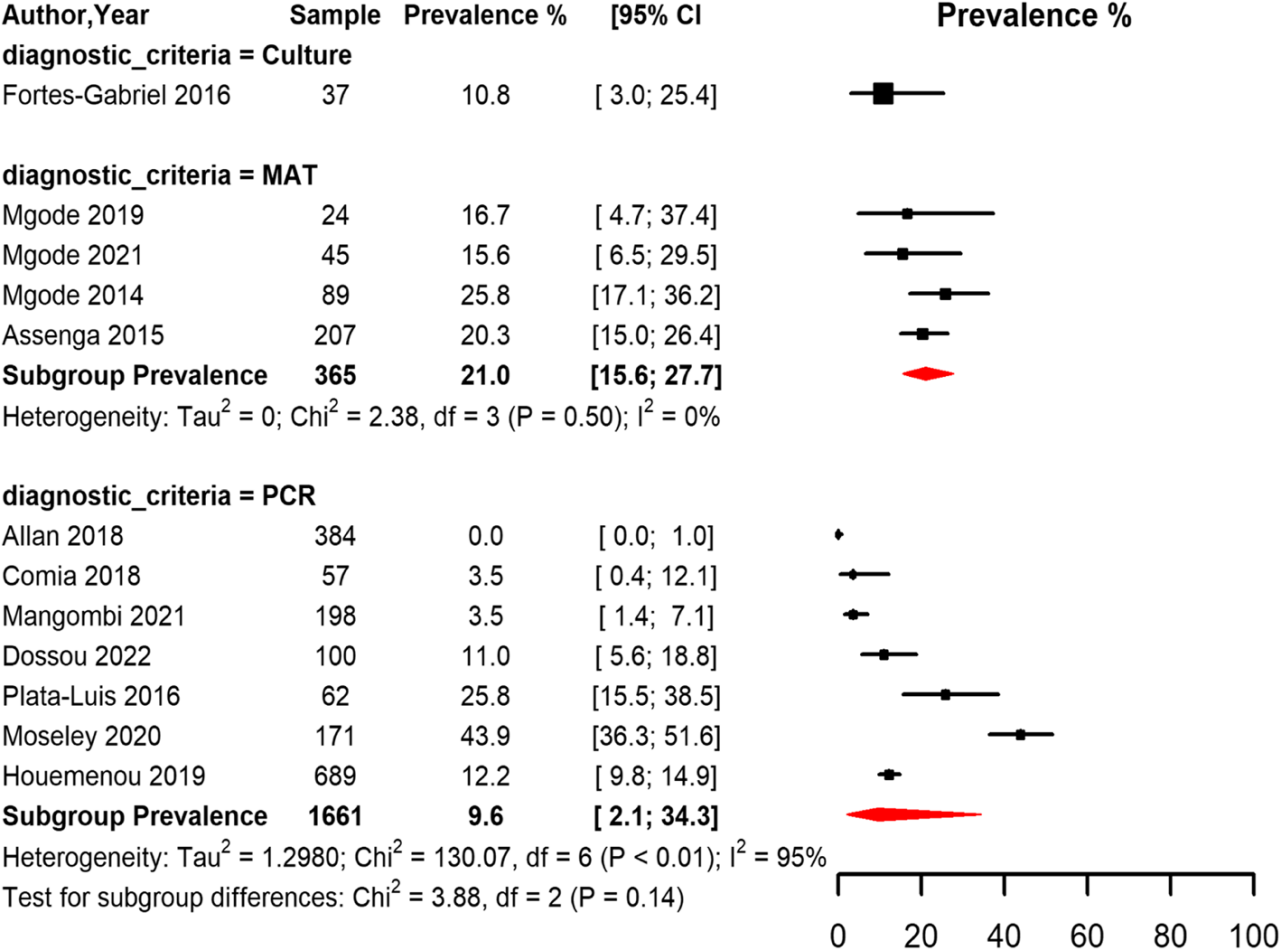
Forest plot of the seroprevalence estimates of Leptospirosis in rodents across SSA regions grouped by the diagnostic method. (MAT: Mat agglutination test; PCR: polymerize chain reaction; CI: confidence interval. The red diamonds represent the pooled prevalence for each diagnostic test)

Among rodents, most of the studies used the PCR method followed by the MAT method, only one study used culturing method and no study used the ELISA method. The pooled seroprevalence estimates of leptospirosis among the studies that used the MAT method was 21.0% (95% CI 15.6,27.7) with no heterogeneity among the pooled studies (*p* < 0.5). Among the studies that used the PCR method, the pooled seroprevalence estimate was 9.6% (95% CI 21.,34.3) with substantial heterogeneity (I^2^ = 95.0%, *p* < 0.01) Fig. 7.

## Discussion

This systematic review and meta-analysis provide the current synthesis and integrated data on the seroprevalence of *Leptospira* infection among humans and selected animal species in SSA. Leptospirosis continues to be among the neglected tropical zoonotic disease and its less prioritized for research and surveillance in most countries in SSA. Yet, this meta-analysis further reveals that the current overall seroprevalence of *Leptospira* infection among humans in SSA is relatively high regardless of the diagnostic method used. *Leptospira* infection among all the selected animals was also higher though it varied based on the diagnostic test used.

Among humans, the overall seroprevalence of *Leptospira* infection in SSA was 12.7%, 15.1%, and 4.5% by ELISA, MAT, and PCR methods respectively. These results fall within the range (2.3% to 19.8%) reported by Kathryn Allan and colleagues in a systematic review synthesizing the prevalence of human leptospirosis in Africa among studies published between 1930 and 2014 [17]. However, the upper confidence limits of the overall prevalence estimated in our meta-analysis (Fig. 3 and 4) was higher than the maximum prevalence (19.8%) reported in the systematic review by Kathryn Allan and colleagues [17]. Our findings reveal that leptospirosis is a recurrent illness and could be significantly contributing to the febrile illness burden in the African region [61]. In addition, the evidence synthesized showed that the prevalence of *Leptospira* infection among humans in SSA was widely spread with varying morbidity based on SSA regions. This finding is consistent with findings from other LMIC and resource-limited settings such as the Caribbeans and Latin America, India, and south-east Asia [62, 63]. Variation in the regional burden of *Leptospira* infection in SSA could be attributed to several factors such as awareness levels, availability of diagnostic facilities, limited resources, climatic and weather differences, and demographic variations.

Studies conducted among animals (cattle, goats, and rodents) were fewer compared to studies conducted among humans. However, a higher pooled prevalence of *Leptospira* infection using the MAT method was estimated in all three species (cattle 30.1%, goats 30.0%, rodents 21.0%). Though these estimates are slightly lower than what has been reported in other tropical or sub-tropical regions [64, 65], they show that the burden of leptospirosis is high and could probably be underestimated because of diagnostic challenges. These findings indicate the importance of *Leptospira* infection on livestock health and production in SSA. This, therefore, demands that future leptospirosis research should prioritize investigating the impact of the *Leptospira* infections on livestock production in the region [66]. Addressing the negative impacts of *Leptospira* infection on livestock production, could directly or indirectly contribute to enhanced human health and well-being in SSA. The high prevalence of *Leptospira* infection among rodents indicates how much of a threat these species are as a sustained reservoir source for human infections [67]. Rodents are implicated as important species in the transmission of *Leptospira* pathogens among humans in urban settings mostly in urban slums [11, 16, 67]. Implementation of rodent control measures would help to curb the transmission of leptospirosis in SSA regions.

A comprehensive understanding of reservoir and carrier animal hosts is essential in the process of deciphering the epidemiology, transmission dynamics, and prevention of leptospirosis both in humans and animals in SSA [4, 17]. In this review, most human studies were conducted independently of the animal studies and among those that sampled both humans and animals simultaneously, a link was not established between human infection and animal infection. Future studies should focus more on establishing the linkage between human and animal *Leptospira* infection within a given study area. Leveraging the One Health approach would aid in effectively quantifying the connection between *Leptospira* infection in humans and animals of importance as well as the role of the environment in the leptospirosis epidemiological triad [17, 68].

### Limitations

The data included in this meta-analysis to a large extent is a tip of an ice bag of leptospirosis morbidity in SSA and therefore it’s not conclusive. Several factors such as limited awareness and paucity of diagnostic facilities likely drive the issues of underreporting of *Leptospira* infection both in humans and animals. Other factors such as over-representation of certain countries or regions such as Tanzania may have contributed to reporting bias, particularly in the spatial distribution of the studies. This, therefore, necessitates that more studies on *Leptospira* infection in humans need to be conducted in CA, WA, and SA regions and some countries in the EA region to explicitly decipher the epidemiology of leptospirosis in SSA. In addition, the level of heterogeneity between the pooled studies was quite high in this review, a challenge common to meta-analyses of prevalence studies. Sub-group analysis based on SSA region (EA, WA, CA, and SA), and study setting (rural and urban) was conducted to ascertain the sources of heterogeneity among studies that involved human participants. However, the heterogeneity persisted, and it could largely be attributed to differences in study participants' characteristics and varying case definitions. Lastly, unpublished data or grey literature were not included in this review, hence some relevant unpublished/ grey literature may have been missed. The synthesized data from animal studies should also be interpreted with caution because most animals in the studies were sampled from an abattoir, and therefore this creates a selection bias since most animals for slaughter tend to be older, and fluctuation in leptospirosis occurrence based on the season of the year was not adjusted for because most studies often did not report this data. Notwithstanding the limitations, this meta-analysis provided a current synthesis of the prevalence of *Leptospira* infection in humans and animals based on diagnostic methods and regions in SSA.

## Conclusion

Leptospirosis continues to remain an important emerging zoonotic disease threatening public health in SSA. This meta-analysis revealed that the overall prevalence of *Leptospira* infections in SSA is high both in humans and animals regardless of the diagnostic method (ELISA or MAT). Upstream factors such as climate change, exponential population increase, expeditious urbanization, and increased interaction between humans and animals are critical in driving the dynamics of leptospirosis occurrence in Sub-Saharan Africa. Prospective leptospirosis research should prioritize the investigation of the interactions between human, animal, and environmental factors and how these interactions drive the leptospirosis burden in SSA. In addition, leptospirosis should be listed among the priority diseases among the diseases causing febrile illnesses for routine seroprevalence and diagnostics to inform timely and appropriate interventions using one health approach.

### Supplementary Information

**Additional file 1.** 

**Additional file 2:** **Supp Table 1.** PubMed main search terms. **Supp Fig 1.** Funnel plot testing for publication bias in studies pooled to estimate the prevalence of leptospirosis among humans in different SSA regions based on the ELISA method. Since the funnel plot is symmetrical, there is no evidence of publication bias. **Supp Fig 2.** Funnel plot testing for publication bias in studies pooled to estimate the prevalence of leptospirosis among humans in different SSA regions based on the MAT diagnostic method. Since the funnel plot is symmetrical, there is no evidence of publication bias. **Supp Fig 3.** Forest plot of the seroprevalence estimates of leptospirosis by ELISA method in humans across setting in SSA. (Setting refers to whether the study was conducted in a rural area, urban area, or a mixture of both urban and rural settings [urban_rural]; CI: confidence interval. The red diagonals represent the pooled prevalence for each study setting and the overall prevalence). **Supp Fig 4.** Forest plot of the seroprevalence estimates of leptospirosis by MAT method in humans across setting in SSA. (Setting refers to whether the study was conducted in a rural area, urban area, or a mixture of both urban and rural settings [urban_rural]; CI: confidence interval. The red diagonals represent the pooled prevalence for each study setting and the overall prevalence). **Supp Table 2.** Pooled seroprevalence of leptospirosis for humans, cattle, goats, and rodents sub-grouped based on the diagnostic criteria.

## Data Availability

All data analyzed during this study are included in this published article [and its supplementary information files].

## Abbreviations

CA: Central Africa
CI: Confidence interval
EA: East Africa
ELISA: Enzyme-linked immunoassay
LMIC: Low-middle-income countries
MAT: Microagglutination test
PCR: Polymerase chain reaction
SA: Southern Africa
SSA: Sub-Saharan Africa
WA: West Africa
