# Supplementary material for: Leptospirosis in humans and selected animals in Sub-Saharan Africa, 2014–2022: a systematic review and meta-analysis
Source: BMC Infect Dis. 2023 Oct 3;23:649. doi: 10.1186/s12879-023-08574-5 (PMC10546638; doi:10.1186/s12879-023-08574-5)
Supplement: Supplementary file 2 — Additional file 2: Supp Table 1. PubMed main search terms. Supp Fig 1. Funnel plot testing for publication bias in studies pooled to estimate the prevalence of leptospirosis among humans in different SSA regions based on the ELISA method. Since the funnel plot is symmetrical, there is no evidence of publication bias. Supp Fig 2. Funnel plot testing for publication bias in studies pooled to estimate the prevalence of leptospirosis among humans in different SSA regions based on the MAT diagnostic method. Since the funnel plot is symmetrical, there is no evidence of publication bias. Supp Fig 3. Forest plot of the seroprevalence estimates of leptospirosis by ELISA method in humans across setting in SSA. (Setting refers to whether the study was conducted in a rural area, urban area, or a mixture of both urban and rural settings [urban_rural]; CI: confidence interval. The red diagonals represent the pooled prevalence for each study setting and the overall prevalence). Supp Fig 4. Forest plot of the seroprevalence estimates of leptospirosis by MAT method in humans across setting in SSA. (Setting refers to whether the study was conducted in a rural area, urban area, or a mixture of both urban and rural settings [urban_rural]; CI: confidence interval. The red diagonals represent the pooled prevalence for each study setting and the overall prevalence). Supp Table 2. Pooled seroprevalence of leptospirosis for humans, cattle, goats, and rodents sub-grouped based on the diagnostic criteria. [file 12879_2023_8574_MOESM2_ESM.docx]

**Title: Leptospirosis in humans and selected animals in Sub-Saharan Africa, 2014-2022: A systematic review and meta-analysis**

Jacob Mugoya Gizamba^1,2^, Lawrence Mugisha^1,3^

*^1^ Department of Wildlife and Aquatic Animal Resources, College of Veterinary Medicine, Animal Resources & Biosecurity, Makerere University, Kampala, Uganda*

*^2^* *Spatial Science Institute, University of Southern California, Los Angeles, United States of America.*

*^3^ Ecohealth Research Group, Conservation &Ecosystem Health Alliance, Kampala, Uganda*

**Supplementary files**

Supp Table 1: PubMed main search terms

| # | Search terms |
| --- | --- |
| 1 | “Africa” OR “Sub-Saharan Africa” |
| 2 | “seroprevalence” OR “prevalence” OR “burden” |
| 3 | “leptospirosis" OR "Leptospira serovars" |
| 4 | #2 AND #3 |
| 5 | #4 AND #1 |

**
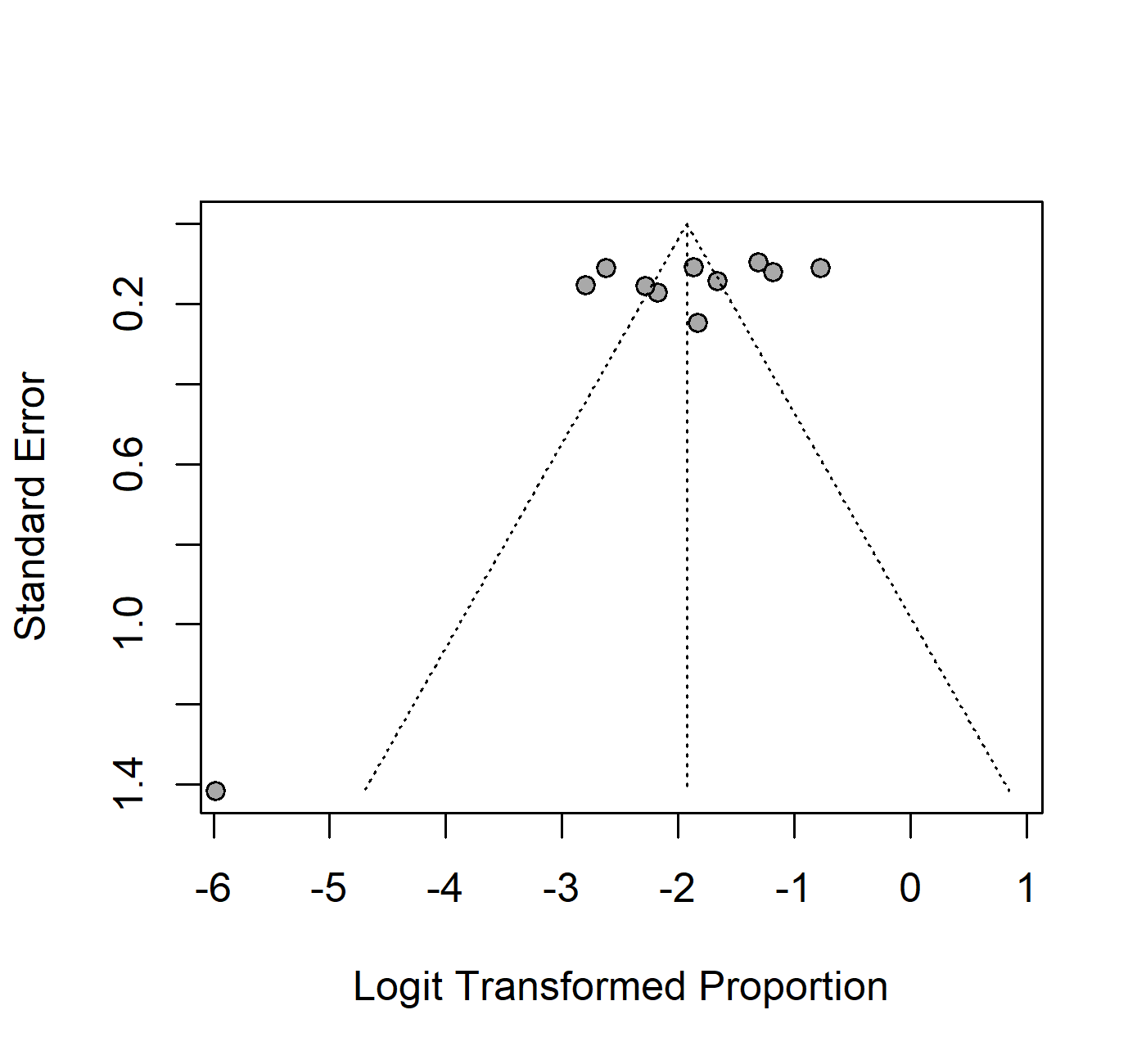
**

Supp Fig 1: Funnel plot testing for publication bias in studies pooled to estimate the prevalence of leptospirosis among humans in different SSA regions based on the ELISA method. Since the funnel plot is symmetrical, there is no evidence of publication bias.


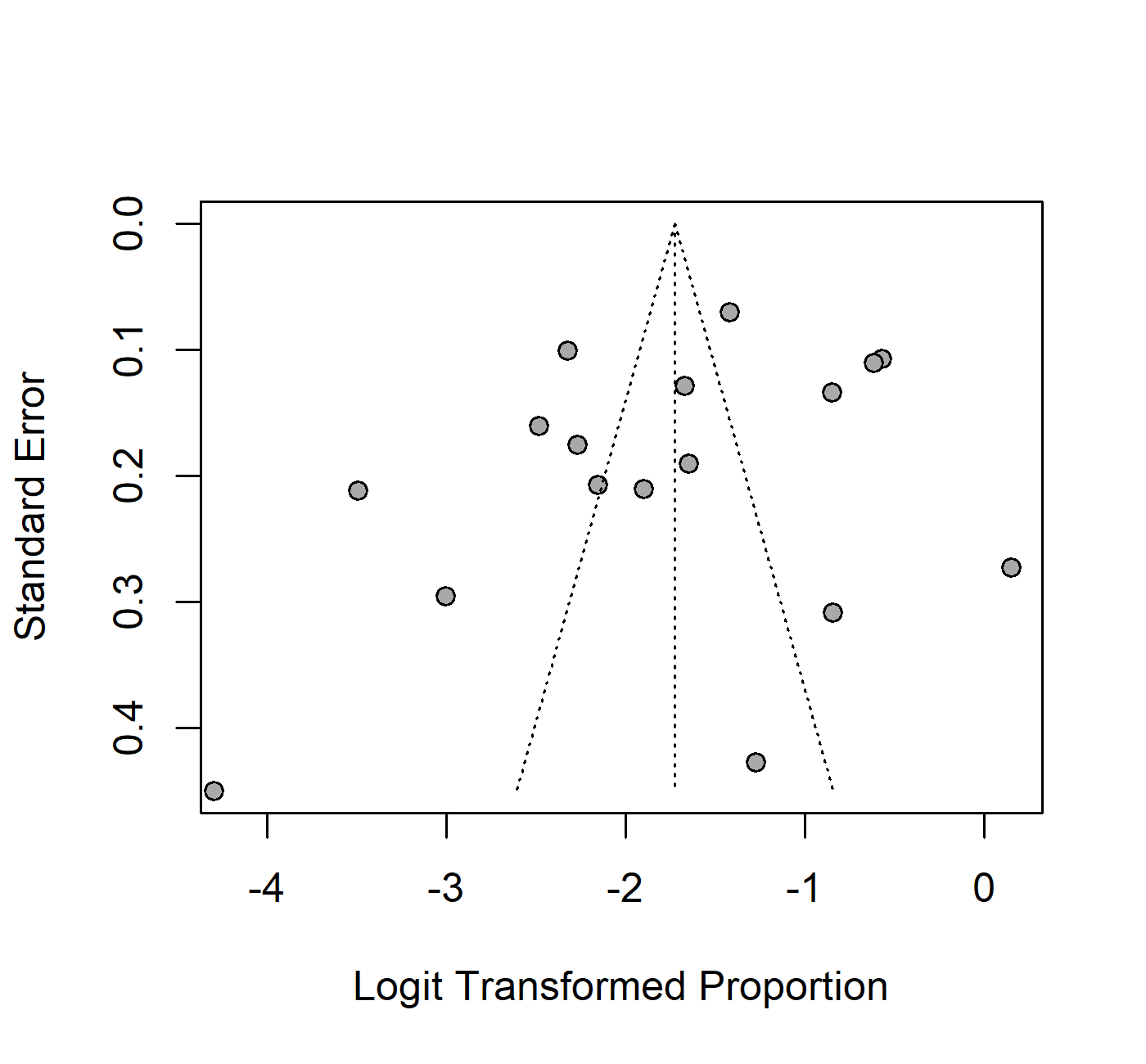


Supp Fig 2: Funnel plot testing for publication bias in studies pooled to estimate the prevalence of leptospirosis among humans in different SSA regions based on the MAT diagnostic method. Since the funnel plot is symmetrical, there is no evidence of publication bias.


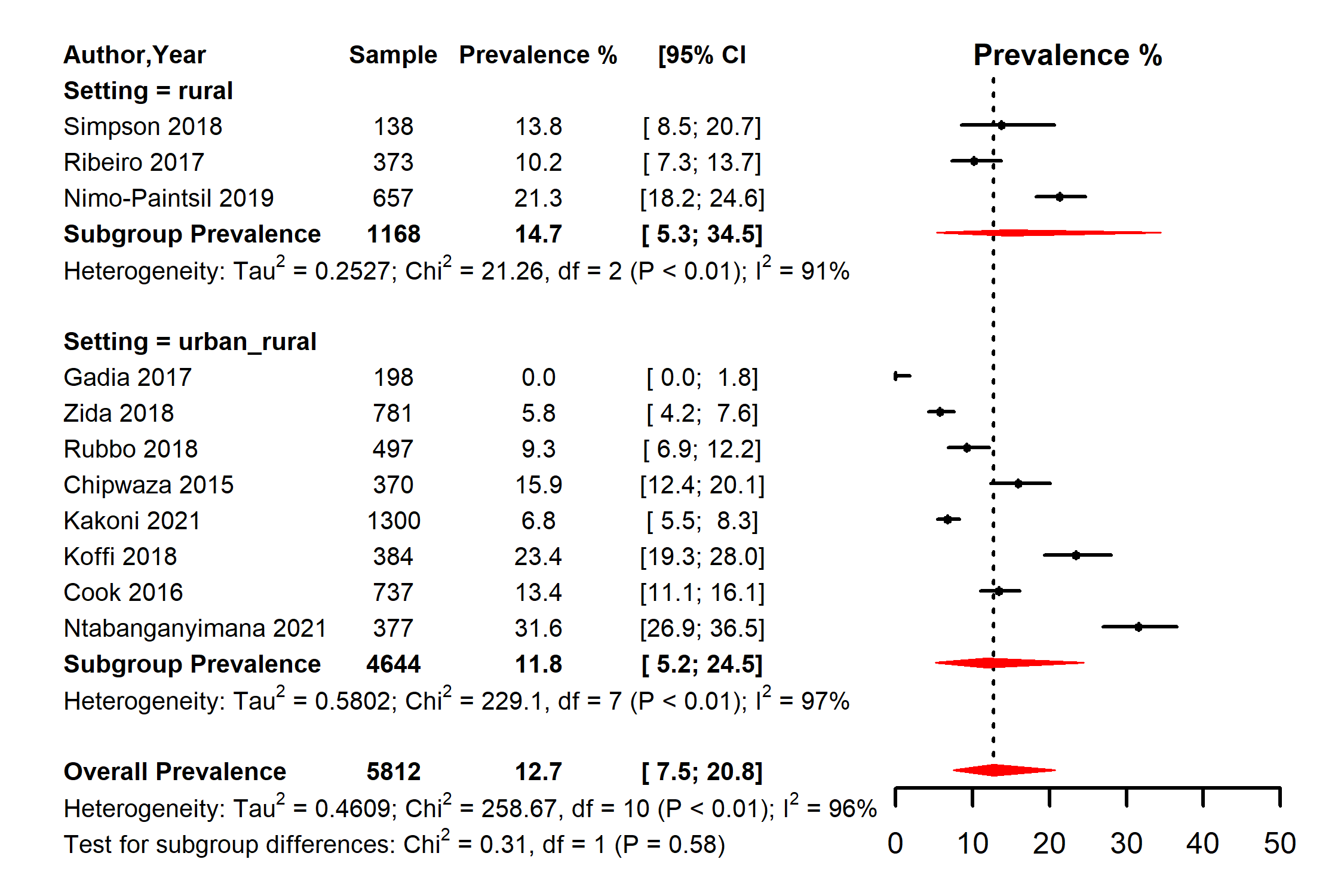


Supp Fig 3: Forest plot of the seroprevalence estimates of leptospirosis by ELISA method in humans across setting in SSA. *(Setting refers to whether the study was conducted in a rural area, urban area, or a mixture of both urban and rural settings [urban_rural]; CI: confidence interval. The red diagonals represent the pooled prevalence for each study setting and the overall prevalence)*


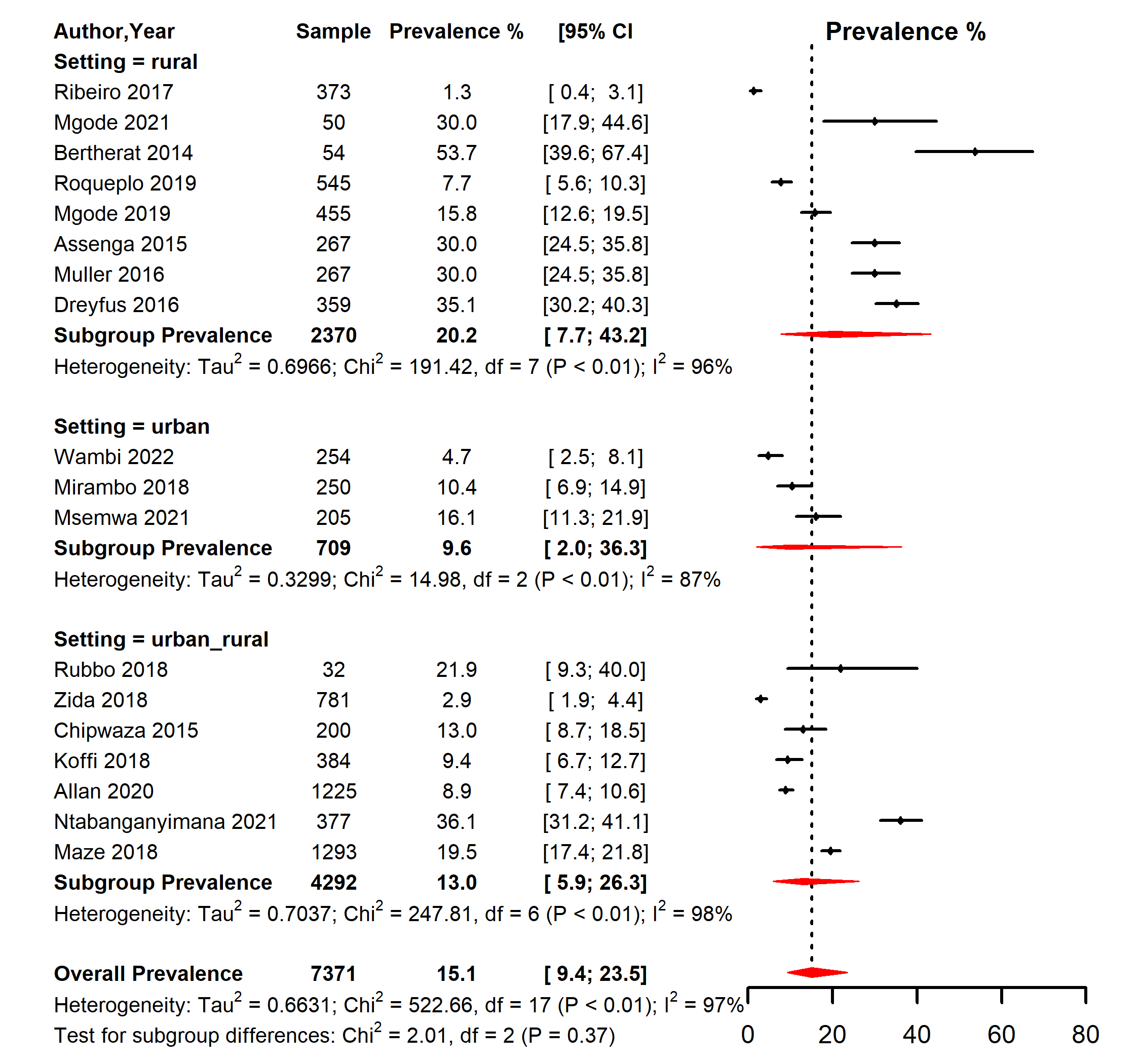


Supp Fig 4: Forest plot of the seroprevalence estimates of leptospirosis by MAT method in humans across setting in SSA. *(Setting refers to whether the study was conducted in a rural area, urban area, or a mixture of both urban and rural settings [urban_rural]; CI: confidence interval. The red diagonals represent the pooled prevalence for each study setting and the overall prevalence)*

Supp Table 2: Pooled seroprevalence of leptospirosis for humans, cattle, goats, and rodents sub-grouped based on the diagnostic criteria.

| Species | Diagnostic criteria | prevalence (95% CI) | # Studies | # Participants | sub-group p-value* |
| --- | --- | --- | --- | --- | --- |
| Human | ELISA | 12.73 (7.51; 20.75) | 11 | 5812 | 0.387 |
|  | MAT | 15.12 (9.37, 23.48) | 18 | 7371 |  |
|  | PCR | 4.47 (0.39,35.60) | 6 | 2080 |  |
| Cattle | ELISA | 29.16 (16.08,46.92) | 3 | 1677 | <0.001 |
|  | MAT | 30.06 (28.02,32.18) | 4 | 1378 |  |
|  | PCR | 9.74 (0.15,88.60) | 2 | 952 |  |
|  | Culture | 76.47 (69.51,82.25) | 1 | 170 |  |
| Goats | MAT | 29.98 (1.15,94.05) | 3 | 345 | 0.001 |
|  | PCR | 1.23 (0.31,4.80) | 1 | 162 |  |
| Rodents | MAT | 21.01 (15.57,27.74) | 4 | 365 | 0.144 |
|  | PCR | 9.64 (2.13,34.33) | 7 | 1661 |  |
|  | Culture | 10.81 (4.12,25.49) | 1 | 37 |  |

*MAT: Mat agglutination test; ELISA: Enzyme-linked immunoassay; PCR: polymerize chain reaction; CI: confidence interval; #: number. * The sub-group p-values indicate whether the pooled seroprevalence differed significantly between the diagnostic methods.*
